# Factors associated with psychotropic drugs use in older adults hospitalized in clinical and surgical wards of a university hospital

**DOI:** 10.1590/1980-5764-DN-2024-0228

**Published:** 2025-07-11

**Authors:** Rafael Thomazi, Carlos Alberto dos Santos, Alessandro Ferrari Jacinto

**Affiliations:** 1 Universidade Estadual Paulista, Botucatu SP Brazil.; 2 Centro Universitário de Adamantina, Departamento de Medicina, Adamantina SP, Brazil.

**Keywords:** Inpatients, Aged, Polypharmacy, Potentially Inappropriate Medication List, Psychotropic Drugs, Pacientes Internados, Idoso, Polimedicação, Lista de Medicamentos Potencialmente Inapropriados, Psicotrópicos

## Abstract

Prescription of psychotropic drugs and potentially inappropriate medications (PIMs) for older adults is a common condition. Assessing the factors associated with this prescription is essential especially within the hospital setting, requiring the implementation of strategies for promoting rational use of medications in this age group.

**Objective:**

To evaluate factors associated with the use of psychotropic drugs in hospitalized older adults.

**Methods:**

Cross-sectional study, conducted at Hospital das Clínicas de Botucatu, Brazil. Patients were divided into clinical patients taking or not taking psychotropic drugs and surgical patients taking or not taking psychotropic drugs. Multivariate analysis was performed, adopting "use or non-use of psychotropic drugs" and "having geriatrician as prescriber before hospitalization or otherwise", as dependent variables.

**Results:**

Of the 385 participants, 60% practiced polypharmacy and 55% were in use of PIM. Clinical patients using psychotropic drugs took more PIM (p<0.001) and more medications (p=0.002) than non-users. For the total sample, PIM use was associated with a 4.53 times greater chance of taking psychotropic drugs, and each additional medication used was associated with a 1.15 times greater chance of taking psychotropic drugs. Being accompanied by a geriatrician before hospital admission was associated with a 4.0 times greater chance of no PIM being prescribed.

**Conclusion:**

PIM use and polypharmacy were associated with an increased chance of taking psychotropic drugs. Being accompanied by a geriatrician before hospitalization was associated with a lower chance of PIM use.

## RESUMO.

A prescrição de psicofármacos e medicamentos potencialmente inapropriados (MPI) para idosos é uma condição comum. Avaliar os fatores associados a essa prescrição é essencial, especialmente no ambiente hospitalar, exigindo a implementação de estratégias para promover o uso racional de medicamentos nessa faixa etária.

**Objetivo:**

Avaliar fatores associados ao uso de psicofármacos em idosos hospitalizados.

**Métodos:**

Estudo transversal, realizado no Hospital das Clínicas de Botucatu, Brasil. Os pacientes foram divididos em quatro grupos: pacientes clínicos que tomavam ou não psicofármacos na admissão hospitalar e pacientes cirúrgicos que tomavam ou não psicofármacos na admissão hospitalar. A análise multivariada adotou como variáveis dependentes o "uso ou não de psicofármacos" e "ter ou não ter geriatra como prescritor antes da internação".

**Resultados:**

Dos 385 idosos, 60% apresentaram polifarmácia e 55%, uso de medicamento potencialmente inapropriado (MPI). Pacientes clínicos que usavam psicofármacos tomavam mais MPI (p<0,001) e mais medicamentos (p=0,002) do que aqueles que não usavam. Na amostra total o uso de MPI aumentou em 4,53 vezes a chance de se tomar psicofármaco, e cada aumento de medicamentos utilizados foi associado a 1,15 vez mais chance de se tomar psicofármaco. Ser acompanhado por um geriatra antes da admissão hospitalar aumentou em quatro vezes a chance de não haver prescrição de um MPI.

**Conclusão:**

O uso de MPI e a polifarmácia aumentaram as chances de se tomar psicofármaco. Ser acompanhado por um geriatra previamente à internação reduziu as chances de se encontrar um MPI.

## INTRODUCTION

Population aging and increased life expectancy are global phenomena^1,2^, accompanied by an increase in the use of medications by the older people population^3-5^.

Lack of vigilance regarding the use of potentially inappropriate medications (PIM) by older patients, including psychotropic drugs, can lead to unfavorable health outcomes in this group^6-8^. The context of a hospital stay can provide an ideal opportunity to perform this assessment and avoid iatrogenic measures involving the misuse of medications^9,10^.

This study aimed to identify factors associated with the use of psychotropic drugs by older adults, as well as the frequency of polypharmacy and PIM use at hospital admission.

## METHODS

This cross-sectional study was carried out at the Hospital das Clínicas de Botucatu located in the state of São Paulo, Brazil. This work was approved by the Ethics Committee of the Faculty of Medicine of Botucatu, on November 13, 2017 (65573417.6.0000.5411). All patients or their guardians signed an informed consent form to participate in the study. The inclusion criteria were older people hospitalized in the selected medical and surgical wards during the period spanning from January 2018 to March 2019. The participants were divided into four groups: G1—clinical ward group not taking psychotropic drugs before hospitalization (n=101), G2—clinical ward group taking psychotropic drugs before hospitalization (n=102), G3—surgical ward group not taking psychotropic drugs before hospitalization (n=102), and G4—surgical ward group taking psychotropic drugs before hospitalization (n=80).

The independent variables investigated were sex, age, education, type of ward in which the patient was hospitalized, presence of polypharmacy and PIM use. Information was also collected on whether the patient was taking psychoactive drugs before hospitalization, including time in use and the prescribing medical specialty, and also whether any new psychotropic drugs were prescribed during hospitalization. Polypharmacy was defined as the use of five or more different medications^11^.

The evaluation of PIM for the elderly was based on the Beers criteria of the American Geriatric Society (AGS Beers Criteria^®^)^12^.

In statistical analyses, continuous variables were expressed as measures of central tendency. Comparisons between means were performed using Student's t-test or variance analysis (ANOVA). Comparisons between medians were performed using the Mann-Whitney or Kruskal-Wallis tests. Associations of categorical variables were determined using the chi-square test. The multivariate analysis employed the logistic regression model. A statistical significance level of 0.05 was adopted.

## RESULTS

The sample comprised a total of 385 patients. Regarding gender distribution, 188 (48%) patients were male and 197 (52%) female. The surgical group taking psychotropics (G4) had twice as many women as men (p=0.003).

The average number of medications used in the total sample of patients was 5.83 (±3.61). Comparing the four groups, clinical users of psychotropic drugs (G2) took more medications than clinical non-users (G1) (p=0.002). Surgical users of psychotropic drugs (G4) took more medications than surgical non-users (G3) (p=0.002). Comparing clinical and surgical non-users of psychotropic drugs (G1+G3), clinical non-users (G1) took more medications (p=0.022).

Polypharmacy was identified in 233 patients (60%). Comparing polypharmacy rates across groups, there was a difference in the distribution of individuals, with a higher proportion of polypharmacy users among clinical ward patients who took psychotropic drugs (G2) (p<0.001). For the overall sample, the rate of PIM use was 55%. Comparing the rate of PIM use in the different groups, there was a difference in the distribution of individuals, with a higher proportion of PIM users in both clinical and surgical users of psychotropic drugs (p<0.001). Overall, the average number of PIM used was 0.91.

Evaluating the groups that took psychoactive drugs (G2+G4), of a total of 182 patients, 56% took antidepressants, 39% benzodiazepines, 27% anticonvulsants and 14% neuroleptics. Of these users, 52% took at least one potentially inappropriate psychoactive drug. There was a statistically significant difference (p=0.014) in the distribution of use between groups G2 and G4, with a higher proportion of G4 patients using potentially inappropriate psychotropic drugs.

Evaluating clinical ward groups only, the multivariate analysis showed that PIM use was associated with a 4.38 times greater chance of taking psychotropic drugs, maintaining the variables age and sex in the model. On the evaluation of surgical ward groups only, the multivariate analysis showed that PIM use was associated with a 5.03 times greater chance of taking psychotropic drugs, while being female was associated with a 2.14 times greater chance. For the evaluation of clinical and surgical ward groups together, the multivariate analysis showed that, adjusting for sex and age, PIM use was associated with a 4.53 times greater chance of taking psychotropic drugs (Table 1).

**Table 1:** Association of potentially inappropriate medication use with psychotropic drug use in all groups.

Independent variables	OR	95%CI	p-value
Age	1.018	0.994–1.041	0.1385
Sex	0.723	0.464–1.126	0.1511
PIM use	4.536	2.904–7.085	<0.001

Abbreviations: OR, odds ratio; CI, confidence interval; PIM, potentially inappropriate medication.

Evaluating clinical and surgical ward groups together, the multivariate analysis showed that, for each additional medication used, there was a 1.15 times greater chance of taking psychotropic drugs. Being male was a protective factor regarding the use of psychotropic drugs (Table 2).

**Table 2:** Association of number of medications used with psychotropic drugs use in all groups

Independent variables	OR	95%CI	p-value
Age	1.013	0.991–1.036	0.2573
Sex	0.612	0.401–0.935	0.0230
Number of medications	1.155	1.086–1.229	<0.001

Abbreviations: OR, odds ratio; CI, confidence interval.

With regard to the evaluation of the prescribing medical specialty, the multivariate analysis showed that a prescribing geriatrician increased the chance of not prescribing a PIM by 4 compared to other prescribing specialties (Table 3).

**Table 3:** Association of potentially inappropriate medication use with prescribing specialty "geriatrician".

Independent variable	OR	95%CI	p-value
Non-prescription of PIM	4.212	1.189–14.925	0.0259

Abbreviations: OR, odds ratio; CI, confidence interval; PIM, potentially inappropriate medication.

## DISCUSSION

Studies investigating the prevalence of and factors associated with psychotropic drugs use in older populations can help identify health risks related to the use of these drugs, highlighting situations and contexts in which their use may pose a danger to the health of this population group^13^.

On hospital admission of older patients, physicians should be alert to the problems of polypharmacy, use of PIM, including psychotropic drugs, and adverse drug reactions (ADRs). ADRs represent a major public health problem in the geriatric population. In a review of European observational studies on ADR epidemiology, approximately 3.6% of all hospital admissions were caused by ADRs and up to 10% of patients in European hospitals experienced an ADR during a hospital stay^14^. Polypharmacy, in the present study, was identified in 60% of the patients. This high rate of polypharmacy corroborates other studies, such as a retrospective observational study of 483 patients performed in an internal medicine ward of a Portuguese hospital^15^. Polypharmacy was present in over 70% of hospitalized patients. Mangin et al.^16^ published an article describing the creation of the "International Group for Reducing Inappropriate Medication and Polypharmacy" that identified the causes and consequences of the use of PIM and polypharmacy, outlining strategies to reduce PIM use.

Sabbor et al.^17^ found that the presence of polypharmacy increased the odds of patients taking PIM by 3.64. Factors associated with higher PIM prescription were number of chronic diseases, insomnia and the prescribing medical specialty. In the wards assessed by the present study, 55% of the older patients admitted having had at least one PIM prescribed. This rate of PIM prescribed by the present study can be compared to European studies^10,18-21^ reporting a PIM prescription prevalence ranging from 20 to 79%. A 2018 systematic review carried out in Australia on the prevalence of PIM use in hospitalized older inpatients that included 47 articles found a prevalence of 53.2 to 89.8%^22^.

Rochon et al.^23^ found that older women took more medications and self-medicated more than their male counterparts, and also consumed more psychotropic drugs. Women are more likely to have greater comorbidities, frailty and adverse drug events. While women tend to adhere less to treatments, they are more open to discussing deprescription than men^23^. In the present study, male gender proved a protective factor for the consumption of psychotropic drugs.

For the wards where patients had been using psychotropic drugs before hospitalization, potentially inappropriate psychotropic drugs use was found in 52% of the patients. This piece of data draws attention to the need to examine how education is structured and how doctors are prepared regarding the safety and knowledge of prescribing psychotropic drugs to older patients. There was a protective factor in relation to not prescribing PIM when the prescriber was a geriatrician, consistent with the specific practices of the specialty.

Antidepressants and benzodiazepines were the most used psychoactive drugs in this study. This data corroborates the findings of another cross-sectional study involving older outpatients treated under the public health system in Sao Paulo city^24^. In a study carried out at a German hospital^25^, the prevalence of older patients treated with psychotropic drugs was higher in the geriatric sector. This higher prescription rate was influenced by the number of chronic diseases and psychiatric disorders. The same study showed that other risk factors for the prescription of psychotropic drugs were being of female gender and oldest old^25^.

In the present study, PIM use increased the chance of taking psychoactive drugs by 4.53 and, with each additional medication consumed, there was a 15% increase in the chances of consuming a psychotropic drug. According to studies in the USA and Europe, the use of multiple medications by patients with depression and anxiety was associated with increased use of psychotropic drugs^19^. Regular assessment is required in older patients who use a high number of medications and have a psychotropic drug in their prescription, evaluating the possibility of "deprescribing" and gradual withdrawal of potentially inappropriate psychotropic drugs^23^. The limitations of the present study included its cross-sectional design and the data collection, which involved only a single university teaching hospital that provided a tertiary service, influencing the profile of the older patients studied.

PIM use and polypharmacy were associated with an increased chance of taking psychotropic drugs. Being accompanied by a geriatrician before hospitalization was associated with a lower chance of PIM use.

## Data Availability

The datasets generated and/or analyzed during the current study are available from the corresponding author upon reasonable request.
